# Factors associated with the serum 25-hydroxyvitamin D levels in adults

**DOI:** 10.1186/s40795-025-01120-3

**Published:** 2025-07-05

**Authors:** Sue-Hsien Chen, Shu-Ching Chen, Yo-Ping Lai, Pin-Hsuan Chen, Kun-Yun Yeh

**Affiliations:** 1 Community Medicine Research Center, Chang Gung Memorial Hospital, Keelung, Taiwan; 2 Department of Nursing Management, Chang Gung Medical Foundation, Taoyuan, Taiwan; 3 School of Nursing, Chang Gung University, Chang Gung University of Science and Technology, Taoyuan, Taiwan; 4 Department of Medical Research, National Taiwan University Hospital, Taipei, Taiwan; 5 Department of Internal Medicine, National Taiwan University Hospital, Taipei, Taiwan; 6 Division of Hemato-oncology, Department of Internal Medicine, Chang Gung Memorial Hospital, Keelung, Taiwan; 7 College of Medicine, Chang Gung University, Taoyuan, Taiwan

**Keywords:** Vitamin D, 25 (OH)D, Taiwan, Community, Predictive factors, Hypovitaminosis D

## Abstract

**Background:**

Hypovitaminosis D has a significant negative impact on general health. Although some factors associated with hypovitaminosis D have been previously reported, they have seldom been analyzed simultaneously in community-based research. This study aims to investigate some potential factors associated with serum 25 (OH)D level, representing a vitamin concentration.

**Methods:**

A cross-sectional study recruiting 4,457 adults, aged over 18 years, was conducted between August 2019 and December 2023. Sociodemographic data were obtained through structured questionnaires, while anthropometric measurements, hemogram results, and biochemical markers were collected from medical records and physical examination findings. A self-reported depression rating score; health beliefs; and health-related quality of life (HRQoL) factors were assessed with questionnaires. Univariate and multivariate analyses were performed.

**Results:**

The mean serum 25(OH)D level was 29.22 ± 10.45 ng/mL. Multivariate linear regression analysis revealed that serum 25(OH)D levels were positively associated with age (β = 0.258, *p* < 0.001), male sex (β = 0.164, *p* < 0.001), high school education level (β = 0.118, *p* < 0.001), smoking exposure (β = 0.044, *p* = 0.011), alcohol consumption (β = 0.150, *p* < 0.001), hemoglobin concentration (β = 0.062, *p* = 0.001), calcium level (β = 0.047, *p* = 0.005), and the mental component score of health-related quality of life (HRQoL) (β = 0.086, *p* < 0.001). Conversely, serum 25(OH)D levels were negatively associated with coffee consumption (β = − 0.045, *p* = 0.002), estimated glomerular filtration rate (β = − 0.105, *p* < 0.001), blood levels of intact parathyroid hormone (β = − 0.184, *p* < 0.001), phosphorus (β = − 0.033, *p* = 0.040), and triglycerides (β = − 0.060, *p* < 0.001). The overall model explained 29.6% of the variance in serum 25(OH)D levels (*R²* = 0.296). There were close correlations between sociodemographic factors, anthropometric variables, hemograms, biochemical data, self-reported depression rating scores, health beliefs, and HRQoL factors.

**Conclusions:**

This study identified several key variables associated with serum 25(OH)D levels in adults. These findings provide important targets for designing health education programs aimed at reducing the prevalence of vitamin D insufficiency. Future community-based research should consider incorporating additional determinants of vitamin D status, including quantified sunlight exposure, dietary intake of vitamin D-rich foods, and supplementation patterns. Accounting for these broader lifestyle and environmental factors may enable subsequent studies to generate more robust insights into the multifactorial nature of vitamin D regulation and inform more effective public health strategies.

**Supplementary Information:**

The online version contains supplementary material available at 10.1186/s40795-025-01120-3.

## Introduction

Vitamin D, a lipid-soluble vitamin with a steroidal structure, regulates the metabolism of calcium and phosphorus and involves cell proliferation and differentiation [1, 2]. Hypovitaminosis D causes impaired calcium absorption that consequently leads to skeletal abnormalities, growth retardation, and the increased risk of hip fractures. Also, hypovitaminosis D is closely associated with chronic illness including hypertension, diabetes mellitus, cardiovascular diseases (CVD), heart failure, peripheral arterial disease, acute myocardial infarction, major depression, various forms of cancer, autoimmune and inflammatory diseases, decreased immune defenses and increased mortality [2–4]. Serum 25-hydroxyvitamin D [25(OH)D] concentrations, the established biomarker for evaluating vitamin D status, are shaped by a complex interplay of demographic, behavioral, physiological, and genetic factors. Age-related declines in cutaneous synthesis capacity and consistent sex-based differences—wherein women exhibit lower serum levels than men—highlight the significance of demographic determinants. Modifiable lifestyle factors, including smoking, physical inactivity, and inadequate dietary vitamin D intake, have been strongly associated with suboptimal vitamin D status. Endogenous synthesis is further influenced by physiological and environmental variables such as skin pigmentation, patterns of solar exposure, use of photoprotective measures (e.g., sunscreen), and latitude of residence. Moreover, genetic polymorphisms in key vitamin D–metabolizing enzymes, alongside impaired hepatic 25-hydroxylation, contribute to the substantial inter-individual variability in serum 25(OH)D concentrations [5–8]. Additionally, personal health beliefs are associated with the risk of hypovitaminosis D. Saeidifard et al. showed that a higher level of self-efficacy, or belief in one’s ability to make positive lifestyle changes, may reduce the risk of Vitamin D deficiency [9]. The integration of healthy behavioral interventions and educational programs may elevate women’s knowledge and prevent hypovitaminosis D [10]. Chen et al. found that perceived benefits correlate with the intention of vitamin D supplementation [11]. Notably, the relationship between vitamin D status and quality of life in subjects with various medical conditions have been addressed [2, 12, 13]. The analysis of NHANES III 1988–1994 showed that patients with a history of CVD had lower vitamin D concentration and experienced physical and emotional symptoms such as fatigue, edema, and sleeping difficulties that limited their psychological and physical functional status and reduced their quality of life. Taken together, a serum vitamin D level may be affected by sociodemographic factors, lifestyle, physical activity, comorbidity status, metabolisms that are related to vitamin D synthesis, and the balance between calcium and phosphorus, as well as personal health beliefs and quality of life.

Therefore, this cross-sectional study recruited adults living in the community and aimed to investigate factors that may correlate with serum vitamin D levels. Based on the predictive factor approach, development of an appropriate health educative lifestyle program may help people reduce the risk of hypovitaminosis D.

## Materials and methods

### Study design and participants

This cross-sectional study enrolled adult residents aged over 18 years, who had been exposed to a health assessment program at Chang Gung Memorial Hospital (CGMH), from August 2019 to December 2023. If the participants had already been diagnosed with one of the following clinical conditions, they were excluded from the study: dementia; pregnancy; active infection; intensive engagement in a weight-loss plan; and regular use of steroids or megestrol acetate. The cognitive ability of each participant was examined, based upon three fact-based questions including the current year, a basic math addition, and the correct day of the week after the one identified. If the participants failed one of these three questions, they were excluded. The purpose, procedures, rights, and confidentiality of this study were explained to the participants. All of them signed informed consents in accordance with the Helsinki Declaration. The study protocol was approved by the Institutional Review Board of the CGMH in Taiwan.

All participants were required to complete a physical examination, laboratory tests, and medical records comprising their present and past histories of illness through one-on-one interviews. Two research nurses simultaneously examined the complete information acquired from each participant. Finally, 4,457 participants (2,873 women and 1,584 men) met the enrollment requirements.

### Data collection

#### Assessment of sociodemographic variables and physical examination

Sociodemographic data, including age, sex, level of educational attainment, smoking exposure, alcohol consumption, and comorbid status assessed by the Charlson comorbidity index (CCI) were collected. The CCI include d17 medical conditions, each assigned a weighted score (from 1 to 6) based on the associated risk of mortality. The total CCI score was calculated by summing the weights of all present conditions. A cut-off points of ≥ 2 was used to indicate the presence of significant comorbidity, and higher CCI scores reflected greater comorbidity burden and risk of mortality [14]. Educational attainment was classified into the following two groups: less than 12 (high school), and more than 12 years (college and above). Marital status was divided into two categories: currently married and currently unmarried (including single, widowed, divorced, or separated). Cigarette smoking was identified if participants were current or former smokers. Alcohol consumption was identified if participants had four drinks or more per week. Betel quid usage was considered affirmative if participants indicated any usage during the previous year. Tea and coffee drinkers were defined if they had regularly drunk tea and coffee for more than three days per week for over five years. Diet style was classified into vegetarian and non-vegetarian. The physical examination data included measurements such as blood pressure, heart rate, height, weight, appearance, vitals, eyes, ears, throat, skin, chest, heart, abdomen, limbs, nerves, reflexes, joints, lymph nodes, genitals, and waist circumference.

#### Laboratory tests

Anthropometric data, including weight, height, and body mass index (BMI) were collected from each participant. Body height and weight were obtained by an automatic height-weight scale to the nearest 0.1 cm and 0.1 kg respectively. Systolic and diastolic blood pressures were measured in a sitting position, twice, after five minutes’ rest, using validated and calibrated electronic sphygmomanometers. The BMI was calculated as weight (kg) divided by the square of height (m). The following laboratory data was collected: hemogram (hemoglobin (Hb), white blood cell count (WBC), platelet count (PLT)), serum levels of 25(OH)D, alanine aminotransferase (ALT), total bilirubin (T-bil), alkaline phosphatase (Alk-P), blood urea nitrogen (BUN), creatinine, calcium, phosphorus, intact parathyroid hormone (iPTH), fasting glucose, glycated hemoglobin (HbA1C), triglyceride (TG), total cholesterol, high-density lipoprotein cholesterol (HDL-C), low-density lipoprotein cholesterol (LDL-C), albumin, C reactive protein (CRP), and insulin resistance, measured by homeostasis model assessment-insulin resistance (HOMA_IR) using an autoanalyzer (Beckman, USA). The estimated glomerular filtration rate (eGFR, ml/min/1.73 m^2^) was calculated using the abbreviated Modification of Diet in Renal Disease Study equation, corrected to a body surface area of 1.73 m^2^ [15]. To collect information in a standardized way, all data were collected consistently by two qualified technicians who had been trained by certified International Society for the Advancement of Kinanthropometry specialists before this study.

Blood samples were collected from participants in the morning after an overnight fast. Serum was separated by centrifugation and used for the analysis of 25-hydroxyvitamin D [25(OH)D], fasting glucose, and lipid profiles. All serum specimens were aliquoted and stored at − 80 °C in designated freezers at the Keelung Chang Gung Community Medicine Research Center, which is equipped with 24-hour temperature monitoring systems to ensure sample integrity. Sample handling and storage followed standardized laboratory protocols. Radio-frequency identification (RFID) technology will be employed throughout the entire process—from collection and transportation to distribution, storage, analysis, and biobanking. The protocol outlines detailed procedures, including timing of collection and transport, container specifications, laboratory processing timelines, storage duration and temperature, and emergency handling of shipments. Development and implementation of the RFID hardware and database system will be carried out in collaboration with the Department of Information Management, Chang Gung University. Collected specimens will be coded and stored in restricted-access freezer areas, monitored by an electronic entry system. All electronic data will be stored on offline computers in a secured information management room, accessible only to authorized personnel. Personally, identifiable information will be encrypted and stored separately, also offline. Access to all data is strictly limited to research staff involved in community medicine studies.

#### Assessment for health belief

According to existing literature [16], the health beliefs model (HBM) questionnaire using a health belief 64-item scale represents the following six dimensions: perceived susceptibility, perceived severity, perceived benefits, perceived barriers, self-efficacy, and cue to action. This scale produced a score from 1 (“strongly disagree”) to 5 (“strongly agree”), with the total sum of scores ranging from 64 to 320. Higher scores reflected stronger beliefs about the impact of the dimension.

#### Assessment of health-related quality of life (HRQoL)

Health-related quality of life (HRQoL) was assessed by the SF-36 questionnaire, which is a generic and multi-dimensional measurement of self-reported health status [17]. It includes 36 questions grouped into eight domains, which include: general health perception (GH), physical functioning (PF), reduction in performing roles resulting from physical problems (role physical [RP]), reduction in performing roles resulting from emotional problems (role emotional [RE]), social functioning (SF), physical pain (bodily pain [BP]), vitality (VT), and mental health (MH). The results can be summarized into two main categories: the physical component summary (PCS) and the mental component summary (MCS). To calculate the summary scores, factor score coefficients from the original SF-36 validation study were applied to generate weighted combinations of the relevant subscales. Final PCS and MCS scores were obtained through T-score conversion, following the guidelines outlined in the official SF-36 scoring manual. Higher scores with a 0 to 100 range indicated better health.

### Assessment for self-reported depression

#### Assessment of depression

The Taiwanese Depression Questionnaire” (TDQ), composed of a 4-point scale with 18 items for the assessment of depression, is a culturally sensitive depression screening inventory [18]. The subjects are guided to rate each item on a scale from 0 to 3 on the basis of “how often you felt the physical and emotional aspects during the past week”. TDQ scores range from 0 to 54. When a cutoff score of 19 is used, it has a good reliability (sensitivity = 0.89, specificity = 0.92), and internal consistency (Cronbach’s α) is 0.94 [18].

#### Expert validation

Five experts were invited (two internists, one family medicine physician, and two senior nursing practitioners who had all practiced for over 18 years), to review the integrity, suitability, and diction of the questionnaires. They conducted a content validity test, in which the content validity index was 0.93. Under the guidance of study nurses trained by the five experts, each participant took approximately 35–45 min to complete the questionnaires, medical records, and physical examinations. Blood samples were collected after overnight fasting.

### Statistical analysis

All data obtained were analyzed using Statistical Package for Social Sciences software, version 25.0 for Windows. Descriptive statistics were computed for sociodemographic variables, anthropometric measurements, laboratory data, HBM, HRQoL, and TDQ. The Kolmogorov Smirnov test for normality was conducted. To analyze the univariate association between the 25 (OH) D level and all variables, the independent *t*-test, Mann–Whitney U test or simple linear regression model, where appropriate, were used. Correlation matrices visualizing correlations among variables were performed using the Pearson correlation coefficient between each pair of variables and were constructed using Statgraphics Centurion version 19 **(**Statgraphics Technologies, Inc. The plains, Virginia, USA**).**

Variables with statistical significance (*p* < 0.05) in the univariate analysis were entered into the multivariate linear regression analysis to identify the independent variables associated with the 25 (OH) D level. A *p*-value < 0.05 was considered statistically significant.

## Results

Table 1 shows the baseline characteristics of 4,457 adults who participated in the study. The mean 25(OH)D level was 29.22 ± 10.45 ng/mL. Nearly two-thirds of the participants were female, with an average age of 56.81 ± 12.66 years. Three-fourths of the participants were younger than 65 years and had graduated high school. 80% of participant were married. One-fifth of participants smoked cigarettes, over two-fifths consumed alcohol, and around one-third drank tea or coffee. Few (3.1%) were vegetarians. The CCI was 1.89 ± 1.41, indicating that the comorbidity severity of this cohort was mild.

**Table 1:** Basic characteristics of 4,457 Taiwanese adult participants

Variables expressed as mean ± SD or number (%)	
**Patient number**	4457 (100)
**25 (OH)D level (ng/mL)**	29.22 ± 10.45
**Sex**	
Male: Female	1584 (35.5):2873 (64.5)
**Age (years)**	56.81 ± 12.66
< 65: ≥65	3390 (76.1):1067 (23.9)
**Educational attainment**	
High school: College and above	3326(74.6):1131 (25.4)
**Marital status**	
Married: Unmarried	3618(81.2):839(18.8)
**Charlson comorbidity index (CCI)**	1.89 ± 1.41
**Diet**	
Non-vegetarian: vegetarian	4320 (96.9): 137 (3.1)
**Cigarette exposure**	
Yes: No	944 (21.2):3513 (78.8)
**Alcohol consumption**	
Yes: No	1940 (43.5):2517 (56.5)
**Betel quid use**	
Yes: No	252(5.7):4205(94.3)
**Drinking tea habit**	
Yes: No	1429(32.1):3028 (67.9)
**Drinking Coffee habit**	
Yes: No	1606(36.0): 2851(64.0)
**BMI (Kg/m** ^**2**^ **)**	24.79 ± 3.90
**Body Weight (Kg)**	63.0 ± 12.24
**Fasting sugar (mg/dL)**	102.62 ± 26.99
**Glycohemoglobin (mmol/mol)**	5.84 ± 0.85
**Insulin**,** mU/L**	8.43 ± 6.69
**HOMA_IR**	2.26 ± 2.53
**Total cholesterol (mg/dL)**	209.20 ± 41.35
**LDL-C (mg/dL)**	125.10 ± 33.79
**HDL-C (mg/dL)**	57.39 ± 15.28
**Triglyceride (mg/dL)**	125.22 ± 184.61
**Hemoglobin (g/dL)**	13.74 ± 1.51
**White blood cell count (x10** ^**3**^ **/mm** ^**3**^ **)**	5.96 ± 1.78
**Platelet count (x10** ^**3**^ **/mm** ^**3**^ **)**	256.76 ± 62.36
**Blood urea nitrogen (mg/dL)**	13.43 ± 5.42
**Creatinine (mg/dL)**	0.77 ± 0.34
**eGFR (mL/min/1.73 m**^**2**^**)**	95.20 ± 25.60
**Aspartate aminotransferase (U/L)**	25.31 ± 22.01
**Alanine aminotransferase (U/L)**	25.66 ± 19.46
**Total bilirubin (mg/dL)**	0.83 ± 0.33
**Alkaline phosphatase (U/L)**	66.17 ± 20.27
**Calcium (mg/dL)**	9.34 ± 0.33
**Phosphorous (mg/dL)**	3.79 ± 0.53
**Albumin (g/dL)**	4.70 ± 0.27
**C-reactive protein (mg/dL)**	2.11 ± 5.22
**Intact parathyroid hormone (pg/mL)**	45.83 ± 23.41
**Health belief model**	229.76 ± 25.86
Perceived susceptibility	25.56 ± 5.87
Perceived severity	28.15 ± 6.11
Perceived benefits	44.11 ± 5.67
Perceived barriers	30.75 ± 15.45
Self-efficacy	65.06 ± 9.88
Cue to action	35.57 ± 5.22
**Health-related quality of life**	
PF	89.74 ± 15.60
RP	84.91 ± 32.45
BP	81.45 ± 21.18
GH	65.07 ± 20.37
VT	68.76 ± 19.91
SF	92.11 ± 13.25
RE	88.40 ± 29.06
MH	74.17 ± 17.63
PCS	52.78 ± 7.10
MCS	51.46 ± 8.68
**Self-rating depression scale score**	6.74 ± 7.18
< 19	3135 (92.8)
≥ 19	320 (7.2)

PF, physical functioning; RP, role physical; BP, bodily pain; GH, general health; VT, vitality; SF, social functioning; RE, role emotional; MH, mental health; PCS, physical component summary; MCS, mental component summary

The mean body mass index (BMI) of the participants was 24.79 ± 3.90 kg/m². The laboratory data were all within the normal ranges, including Hb, WBC, PLT, levels of ALT, T-bil, Alk-P, BUN, creatinine, eGFR, calcium, phosphorus, iPTH, albumin, CRP, fasting glucose, HbA1C, insulin, HOMA_IR, TG, HDL, LDL-C, except for mildly elevated total cholesterol (209.20 ± 41.35 mg/dL). The overall scores of HBM, MCS, PCS were 229.76 ± 25.86, 52.78 ± 7.10 and 51.46 ± 8.68, respectively. Regarding the self-rating depression scale total score, 7.2% of participants obtained a score over 19, indicating that they were depressed.

**Fig. 1:**
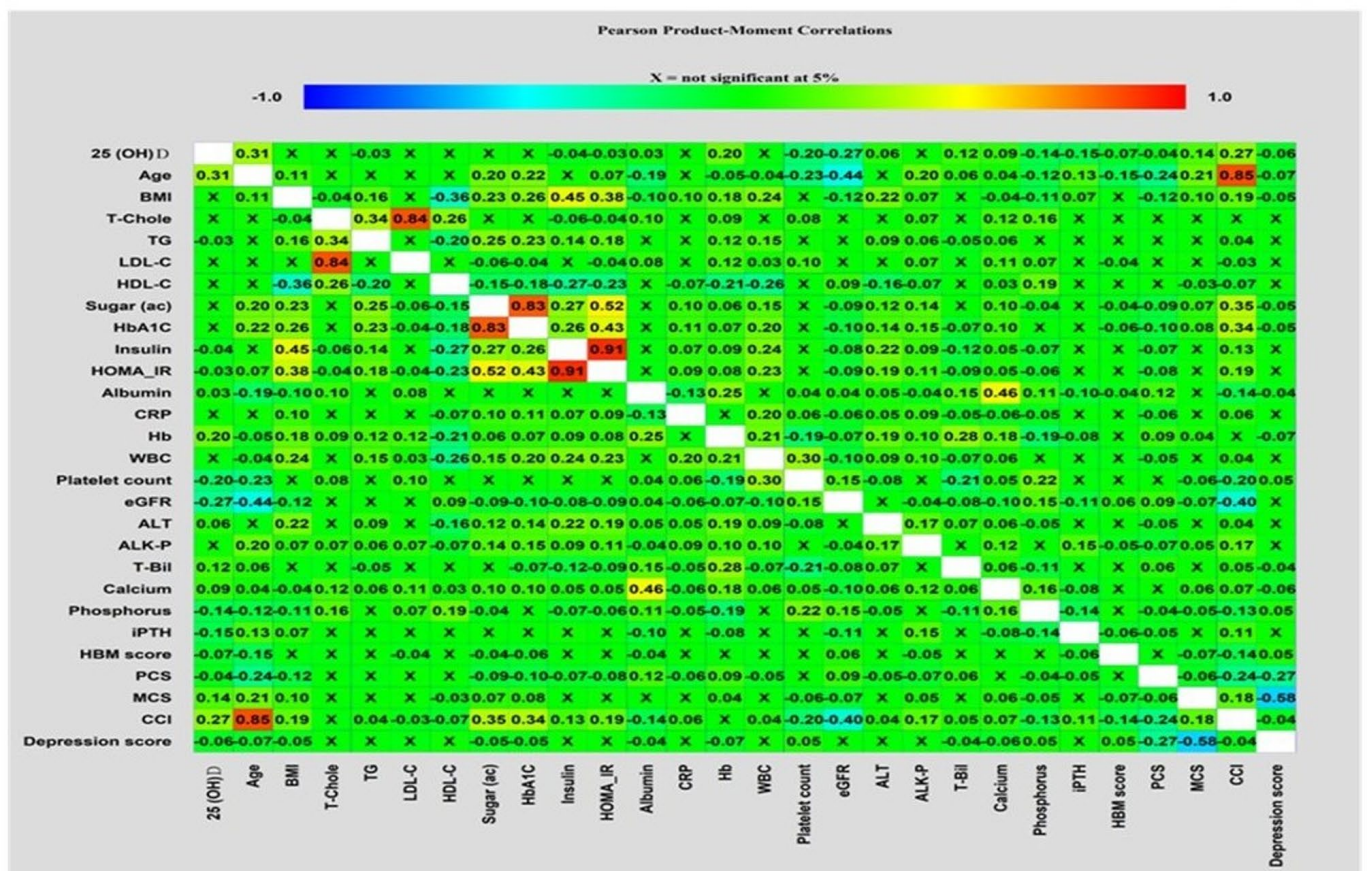
Correlation matrix of variables associated with serum 25(OH)D levels in 4,457 Taiwanese adult participants

Figure 1 shows the relationship between serum levels of 25 (OH)D, age, CCI, anthropometric measurements, laboratory data, HBM, PCS, MCS, and the self-rating depression scale scores. The 25 (OH)D level was positively correlated with age, albumin, Hb, ALT, T-bil, calcium, PCS, MCS, and CCI, and negatively correlated with TG, insulin, HOMA_IR, PLT, eGFR, phosphorus, iPTH, the HBM score and depression scores in the univariate association analysis. Male participants who had a high school qualification, were married, non-vegetarian, smoked cigarettes, consumed alcohol, drank tea, but not coffee, and obtained a depression score < 19, were associated with higher 25 (OH)D levels than their counterparts (Table 2). Multivariate analysis further revealed that a serum 25 (OH)D concentration was positively associated with age, male sex, a high school education level, exposure to smoking and alcohol, Hb, calcium level and MCS of HRQoL, but negatively associated with coffee drinking, blood TG, eGFR, iPTH and phosphorus (Table 3).

**Table 2:** Univariate analysis of the serum 25 (OH)D level in 4,457 Taiwanese adult participants stratified by different sociodemographic variables and self-rating depression scale scores

Variables expressed as mean ± SD	25 (OH)D level (ng/mL)	P*
Sex		
Male vs. Female	33.54 ± 11.47 vs. 26.84 ± 9.01	< 0.001
Education		
High school vs. College and above	30.35 ± 10.46 vs. 25.86 ± 9.70	< 0.001
Marital status		
Married vs. Unmarried	29.52 ± 10.38 vs. 27.90 ± 10.68	< 0.001
Diet		
non-vegetarian vs. vegetarian	29.51 ± 10.33 vs. 20.17 ± 10.48	< 0.001
Smoking		
Yes vs. No	32.61 ± 11.75 vs. 28.30 ± 9.84	< 0.001
Alcohol		
Yes vs. No	30.56 ± 11.06 vs. 28.19 ± 9.84	< 0.001
Tea		
Yes vs. No	29.68 ± 10.51 vs. 29.00 ± 10.42	0.047
Coffee		
Yes vs. No	27.88 ± 9.96 vs. 29.97 ± 10.65	< 0.001
depression score		
< 19 vs. ≥19	29.31 ± 10.47 vs. 27.99 ± 10.24	0.033

*P < 0.05, statistical significance

**Table 3:** Multivariate associations of variables correlated with serum 25 (OH)D level in 4,457 adult participants

Variable	Coefficient	95% CI	P*
Age	0.258	0.166 to 0.264	< 0.001*
Gender (ref: female)	0.164	2.670 to 4.491	< 0.001*
Educational attainment (ref: College and above)	0.118	1.800 to 3.340	< 0.001*
Marital status (ref: Married)	0.016	-0.329 to 1.205	0.262
Smoking (ref: No)	0.044	0.187 to 1.926	0.011*
Alcohol consumption (ref: No)	0.150	1.518 to 2.782	< 0.001*
Betel use (ref: No)	0.023	-0.352 to 2.484	0.140
Tea (ref: No)	0.000	-0.631 to 0.664	0.985
Coffee (ref: No)	-0.045	-1.601 to -0.363	0.002*
CCI	-0.009	-0.429 to 0.378	0.746
Insulin	-0.007	-0.117 to 0.095	0.838
HOMA_IR	-0.055	-0.509 to 0.048	0.104
Triglyceride	-0.060	-0.005 to -0.002	< 0.001*
Hemoglobin	0.062	0.181 to 0.682	0.001*
eGFR	-0.105	-0.056 to -0.030	< 0.001*
Alanine aminotransferase	0.024	-0.003 to 0.028	0.105
Total bilirubin	0.002	-0.894 to 1.040	0.882
Calcium	0.047	0.356 to 2.458	0.005*
Phosphorous	-0.033	-1.274 to -0.029	0.040*
Intact parathyroid hormone	-0.184	-0.097 to -0.071	< 0.001*
Albumin	0.007	-1.006 to 1.532	0.685
Depression score (ref: <19)	0.029	-0.106 to 1.438	0.341
Health belief	-0.009	-0.015 to 0.008	0.555
PCS	0.015	-0.023 to 0.067	0.339
MCS	0.076	0.053 to 0.132	< 0.001*

*P < 0.05, statistical significance

CI, confidence interval; CCI, Charlson comorbidity index; eGFR, estimated glomerular filtration rate; PCS, physical component summary; MCS, mental component summary

## Discussion

The factors influencing serum Vitamin D level are classified into three categories: sociodemographic variables, lifestyle and vitamin D-related metabolisms [5–7, 19–24]. In this study, a vitamin D level, presented as a 25 (OH) D level, had positive associations with age, male sex, high school education level, Hb, calcium level and MCS, but negative associations with coffee drinking, blood TG, eGFR, iPTH and phosphorus. Old age, male sex and high school attainment are consistent with the findings of previous studies have been well discussed [4, 7, 11]. Other factors, classified into four groups: habits of cigarette smoking, alcohol consumption and coffee drinking, nutrition variables (TG, Hb), vitamin D-regulating elements (eGFR, iPTH, calcium, phosphorus) and quality of life (MCS) deserve further discussion as follows.

The circulating 25 (OH)D level is lower in smokers than non-smokers and vitamin D supplementation may avoid more smoking-induced lung tissue damage [24–28]. The current study, however, showed a positive correlation between smoking and serum 25 (OH)D levels. Some postulates are proposed. Smoking may reduce the production of 1, 25-dihydroxyvitamin D, which subsequently may feedback an increased serum substrate 25 (OH)D level [29]. It may also affect the expression of the vitamin D receptor [30], which may modulate the circulating 25 (OH) D concentration. Furthermore, over half of the male participants smoked (55.2%) and nearly one fourth of the entire cohort were aged over 65, factors which may have both contributed to higher vitamin 25 (OH) D levels. Similarly, alcohol may interfere with the absorption of vitamin D, reduce concentrations of vitamin D binding protein, decrease the formation of 25 (OH)D in the liver, alter bile excretion, impair renal function and degrade 1, 25 dihydroxyvitamin D [31–34]. However, some reports have shown that excessive alcohol use may increase serum 25 (OH) D concentrations since alcohol may impede the conversion of 25 (OH) D to 1, 25 dihydroxyvitamin D. Therefore the 25 (OH)D concentration was increased, correlating positively with alcohol use [35, 36]. A meta-analysis tends to favor the protective role of alcohol in reducing the risk of hypovitaminosis D [23]. The increased 25 (OH) D level of alcohol users may be that people often eat protein-rich food and wine pairing when drinking alcohol, thus gaining vitamin D supplementation.

The associations between tea and coffee consumption and vitamin D status remain unclear [7, 20, 37]. Our results indicated that drinking coffee was negatively associated with 25 (OH)D levels. This was in partial accordance with previous reports showing the inhibitory effect of caffeine on vitamin D receptor expression [38]. A reverse correlation was found between a dose of caffeine and 25 (OH)D levels [39]. The discrepancies among studies were likely to be due to variation in doses, brands, frequency and duration of consumption, enrolled population and target diseases.

Hypovitaminosis D is associated with an impaired blood lipid profile including increasing TG, total cholesterol, LDL-C and reduced HDL-C. This correlation may be partially explained in terms of the regulation of lipid metabolism including the inhibition of lipogenesis and increasing lipolysis via vitamin D-mediated calcium spillage into fat cells and the pancreatic islet function [40–43]. Also, hypovitaminosis D is associated with an increased risk of anemia, hypoferritinemia, iron deficiency, reduced serum erythropoietin and low Hb levels [44–46], since vitamin D may directly stimulate the erythropoietin precursor, downregulating circulating hepcidin levels that are increased during inflammation to block iron absorption [44, 47, 48]. Nonetheless, dietary record, the status of vitamins and mineral elements involving lipid profile and hemoglobin synthesis were not included in this analysis. The relationship between 25 (OH)D, TG and Hb should be interpreted with caution.

The serum 25 (OH)D level is also affected by parathyroid hormone, levels of calcium and phosphorus, 1,25-dihyroxyvitamin D concentration and the activities of enzymes involved in vitamin D metabolism [24]. The current study found that the serum 25 (OH)D level had a positive correlation with calcium concentration, and negative correlations with eGFR, levels of iPTH and phosphorus. Although similar results were reported [49, 50] and the positive correlation between 25 (OH)D and calcium was explained [51], the possible mechanisms whereby 25 (OH) D interacts with these negatively correlated factors merits more discussion. The major mechanisms explaining the inverse relationship between 25 (OH)D and iPTH is that 25 (OH)D may enter the chief cells of parathyroid tissue via a free circulating form by concentration-dependent diffusion or bound to vitamin D binding proteins by megalin or cubulin-mediated rapid transport [52, 53]. In the chief cells, the 25 (OH)D and 1,25-dihyroxyvitamin D that is converted from 25 (OH)D by CYP27B1 enzyme may interact with the nuclear vitamin D receptor and inhibit the transcription of iPTH mRNA synthesis [50, 54, 55]. Additionally, a high circulating concentration of 25 (OH)D might inhibit the reuptake and degradation of iPTH at the proximal tubule of the kidney [56]. Our data revealed a negative correlation between eGFR and the iPTH level (Fig. 1), indirectly supporting the involvement of renal function in the vitamin D level. Last but not least, the level of fibroblast growth factor 23 (FGF23), one of the key regulators in iPTH synthesis and serum phosphorus concentrations [57], may modulate 1-α hydroxlase activity and subsequently affects the reabsorption and excretion of calcium, phosphorous and iPTH function in the kidney [50]. In agreement with the effect of FGF23 on iPTH and phosphate, our data showed an association of iPTH with phosphate (Fig. 1). Hence, FGF23 may play a role in the interactions between 25 (OH)D, iPTH, renal function, calcium and phosphorus.

The current study examines the association between participants’ health beliefs, self-report depression statuses and quality of life, with serum 25 (OH)D levels, and found that MCS of HRQoL had an independent and positive association in the multivariate analysis (Table 3). In particular, all four items (VT, SF, RE and MH) of MCS were significantly associated with the 25 (OH)D level (Supplementary Table 1). Feng et al. reported that vitamin D insufficiency defined as a 25 (OH)D level < 30 ng/mL had lower scores of VT, SF and MH of MCS than those with sufficient vitamin D [58]. The other cross-sectional study showed significant associations between vitamin D and HRQoL, particularly in relation to depression and anxiety [59]. The necessity of integrating HRQoL into a healthcare program aimed at preventing hypovitaminosis D.

Certain limitations are acknowledged. Caution in data interpretation is warranted when it comes to extrapolating or generalizing our findings as the participants were voluntarily seeking medical assistance and were a relatively healthy subpopulation. Those who stay in a nursing home, live alone with no assistance, or are unwilling to go out, may have more severe comorbid illnesses, vary sociodemographically, differ in their health beliefs, and quality of life, and may consequently present different factors in relation to serum 25 (OH)D predictions. Also, the cause-and-effect relationships regarding the current associations cannot to be identified in the cross-sectional study. It is important to acknowledge that the data collection period spanned nearly three seasons, which may have introduced variability in serum 25(OH)D levels due to seasonal changes in sunlight exposure. Previous research has demonstrated that vitamin D levels tend to be lower during the winter months, primarily due to reduced ultraviolet B (UVB) radiation. In the present study, however, our analysis of seasonal variation showed no statistically significant difference in serum 25(OH)D levels between the summer–fall period (April to September) and the winter–spring period (October to March), with mean levels of 30.01 ± 10.03 and 29.44 ± 10.92, respectively (*p* > 0.05). This lack of seasonal variation may be partially explained by: (1) Taiwan’s geographic location within the tropics, where sunlight exposure remains relatively consistent throughout the year; and (2) the absence of detailed data on sun exposure and outdoor activity in the present study. Nevertheless, it remains possible that seasonal effects and variations in outdoor activity could have introduced residual confounding. To mitigate the potential influence of these factors, future studies might consider limiting sample collection to a narrower time frame, such as during the winter months. Lastly, as many confounding variables as possible were adjusted for, but the confounding effects of dietary record, inflammatory status, currently used medications, outdoor physical activity, sun exposure, seasonal changes, serum levels of FGF23 and 1,25-dihyroxyvitamin D were not excluded.

## Conclusion

Public health messages on the importance of vitamin D, its role in overall wellbeing and ways of achieving sufficient vitamin D status should be disseminated to the public since hypovitaminosis D has a significant negative impact on the healthcare of society. In this study, factors associated with serum 25 (OH)D levels included sociodemographic variables (age, sex and educational attainment), habits of cigarette smoking, alcohol consumption and coffee drinking, nutrition index (TG, Hb), regulators in vitamin D metabolism (eGFR, iPTH, calcium and phosphorus) and quality of life (MCS). It is believed that a prospective study analyzing all community comers from different health situations, and all potential co-variates is warranted to determine the causal relationship between serum a 25 (OH)D level and these predictive factors. Importantly, local healthcare administrations should pursue these potential factors and tailor their health promotion programs to improve serum 25 (OH) levels and reduce the risk of vitamin D insufficiency This application underlines the necessity of understanding the factors associated with vitamin D status as a public health issue.

## Electronic supplementary material

Below is the link to the electronic supplementary material.

Supplementary Material 1

## Data Availability

The datasets used and analyzed during the current study are available from the corresponding author on reasonable request.

## Abbreviations

AC: Fasting
BP: Bodily Pain
CGMH: Chang Gung Memorial Hospital
CRP: C Reactive Protein
CVD: Cardiovascular Diseases
FBG: Fasting Blood Glucose
GH: General Health Perception
HbA1C: Glycated Hemoglobin
HBM: Health Belief Model
HDL-C: High-Density Lipoprotein Cholesterol
HRQoL: Health-Related Quality of Life
LDL-C: Low-Density Lipoprotein Cholesterol
MCS: Mental Component Summary
MetS: Metabolic Syndrome
MH: Mental Health
NCEP-ATP III: National Cholesterol Education Program-Adult Treatment Panel III
PCS: Physical Component Summary
PF: Physical Functioning
RE: Role Emotional
RP: Role physical
SF: Social Functioning
TG: Triglycerides
VDI: Vitamin D Insufficiency
VT: Vitality
25 (OH)D: 25-hydroxyvitamin D
