# Supplementary material for: Factors associated with the serum 25-hydroxyvitamin D levels in adults
Source: BMC Nutr. 2025 Jul 5;11:132. doi: 10.1186/s40795-025-01120-3 (PMC12228237; doi:10.1186/s40795-025-01120-3)
Supplement: Supplementary file 1 — Supplementary Material 1 [file 40795_2025_1120_MOESM1_ESM.docx]

**Supplementary Table 1. Correlational analysis of the relationship between the serum 25 (OH)D levels and the health-related quality of life of 4,457 adult participants**

|  | **25 (OH)D** | **PF** | **RP** | **BP** | **GH** | **VT** | **SF** | **RE** | **MH** | **PCS** | **MCS** |
| --- | --- | --- | --- | --- | --- | --- | --- | --- | --- | --- | --- |
| **25 (OH)D** |  | **-0.021** | **-0.031** | **0.023** | **0.070*** | **0.091**** | **0.072*** | **0.090**** | **0.085**** | **-0.040*** | **0.140**** |
| **PF** | **-0.021** |  | **0.395**** | **0.459**** | **0.303**** | **0.244**** | **0.281**** | **0.263**** | **0.225**** | **0.730**** | **0.092**** |
| **RP** | **-0.031** | **0.395**** |  | **0.403**** | **0.357**** | **0.331**** | **0.427**** | **0.501**** | **0.305**** | **0.707**** | **0.306**** |
| **BP** | **0.023** | **0.459**** | **0.403**** |  | **.341**** | **0.292**** | **0.340**** | **0.279**** | **0.288**** | **0.728**** | **0.196**** |
| **GH** | **0.077*** | **0.303**** | **0.357**** | **0.341**** |  | **0.615**** | **0.370**** | **0.328**** | **0.575**** | **0.523**** | **0.542**** |
| **VT** | **0.092**** | **0.244**** | **0.331**** | **0.292**** | **0.615**** |  | **0.362**** | **0.335**** | **0.722**** | **0.304**** | **0.744**** |
| **SF** | **0.070*** | **0.281**** | **0.427**** | **0.340**** | **0.370**** | **0.362**** |  | **0.369**** | **0.458**** | **0.340**** | **0.560**** |
| **RE** | **0.021** | **0.263**** | **0.501**** | **0.279**** | **0.328**** | **0.335**** | **0.369**** |  | **0.382**** | **0.204**** | **0.685**** |
| **MH** | **0.085**** | **.225**** | **0.305**** | **0.288**** | **0.575**** | **0.722**** | **0.458**** | **0.382**** |  | **0.165**** | **0.877**** |
| **PCS** | **-0.021** | **.730**** | **0.707**** | **0.728**** | **0.523**** | **0.304**** | **0.340**** | **0.204**** | **0.165**** |  | **0.021** |
| **MCS** | **0.092**** | **.092**** | **0.306**** | **0.196**** | **0.542**** | **0.744**** | **0.560**** | **0.685**** | **0.877**** | **0.021** |  |

**P* value < 0.05 and ** *P* value < 0.01denote statistical significance.

25 (OH)D, 25-hydroyxyvitamin D; PF, physical functioning; RP, role physical; BP, bodily pain; GH, general health; VT, vitality; SF, social functioning; RE, role emotional; MH, mental health; PCS, physical component summary; MCS, mental component summary
